# Dual Effect of Acetic Acid Efficiently Enhances Sludge-Based Biochar to Recover Uranium From Aqueous Solution

**DOI:** 10.3389/fchem.2022.835959

**Published:** 2022-02-22

**Authors:** Shoufu Yu, Xiaoyan Wu, Jian Ye, Mi Li, Qiucai Zhang, Xiaowen Zhang, Chunxue Lv, Wenjie Xie, Keyou Shi, Yong Liu

**Affiliations:** ^1^ University of South China, Hengyang, China; ^2^ Hengyang Key Laboratory of Soil Contamination Control and Remediation, University of South China, Hengyang, China; ^3^ Key Laboratory of Radioactive Waste Treatment and Disposal, University of South China, Hengyang, China; ^4^ Decommissioning Engineering Technology Research Center of Hunan Province Uranium Tailings Reservoir, University of South China, Hengyang, China

**Keywords:** uranium, uranium-containing wastewater, excess sludge, acetic acid, sludge-based biochar

## Abstract

Excess sludge (ES) treatment and that related to the uranium recovery from uranium-containing wastewater (UCW) are two hot topics in the field of environmental engineering. Sludge-based biochar (SBB) prepared from ES was used to recover uranium from UCW. Excellent effects were achieved when SBB was modified by acetic acid. Compared with SBB, acetic acid-modified SBB (ASBB) has shown three characteristics deserving interest: 1) high sorption efficiency, in which the sorption ratio of U(VI) was increased by as high as 35.0%; 2) fast sorption rate, as the equilibrium could be achieved within 5.0 min; 3) satisfied sorption/desorption behavior; as a matter of fact, the sorption rate of U(VI) could still be maintained at 93.0% during the test cycles. In addition, based on the test conditions and various characterization results, it emerged as a dual effect of acetic acid on the surface of SBB, i.e., to increase the porosity and add (−COOH) groups. It was revealed that U(VI) and −COO− combined in the surface aperture of ASBB *via* single-dentate coordination. Altogether, a new utilization mode for SBB is here proposed, as a means of efficient uranium sorption from UCW.

## Introduction

Uranium-containing wastewater (UCW) contains a certain concentration of nuclide ions, such as uranium ions, radium ions, and thorium ions. In addition, it also contains high concentrations of heavy metal ions, metal ions, and acid ions, such as sulfate ions and nitrate ions. This special industrial wastewater is mainly discharged by uranium mining or uranium hydrometallurgy. Generally, uranium presents in the valence form of U(IV) and U(VI). U(IV) does not dissolve in aqueous solution and usually forms precipitation, while U(VI) generally gives mobile aqueous complexes with CO_3_^2−^ and OH^−1^ (Gerber et al., 2016; Abdi et al., 2017). Uranium ions are characterized by radioactivity and chemical toxicity, which could, in turn, cause chronic poisoning, cancer, and immunological diseases (Malenchenko et al., 1978; Baur et al., 1996; Kathren and Burklin, 2008; NaserHumood, 2013). In addition, serious damage might be caused to the surrounding organisms as well as ecosystems once UCW is discharged into the environment by accident. Consequently, it is of great significance to establish rapid and efficient processing methods for UCW treatment aimed at both reducing the hazardous effect of UCW and reusing, as added-value product, uranium recovered by UCW.

At present, the main technologies for uranium recovered from UCW or UCW treatment could be summarized as chemical precipitation, ion exchange, membrane separation, biological treatment, solvent extraction, and sorption (Khani et al., 2008; Wang et al., 2009; Abadi et al., 2011; Gerber et al., 2016; Khawassek et al., 2018; Tan et al., 2018). Among them, sorption is one of the popular technologies because of its advantages, including simple operation, wide range of application, higher removal and recovery rate, etc. (Chen et al., 2020). Generally, the uranium removal rate by sorption is mainly influenced by physical/chemical properties of adsorbents (i.e., pore structure, surface groups), uranium concentration, pH, etc. (Kataria and Garg, 2018) Biochar (Sun et al., 2013; Sun et al., 2017; Kong et al., 2020), graphene (Zhao et al., 2019), calixarene (Fang-Zhu et al., 2019), MOFs (Li et al., 2020), and mesoporous silicon (Jiang et al., 2020) were used as sorption materials. Among them, biochar has been verified as an important sorption material for uranium recovery or removal from aqueous solution (Jin et al., 2018; Li et al., 2019a), due to its simple preparation process, lower price, higher temperature resistance, radiation resistance, higher stability to almost all kinds of acidic and alkali environments, nontoxic, and environmentally friendly nature (Zhao et al., 2017; Pu et al., 2019).

Generally, excess sludge (ES) was applied for biochar preparation (Li et al., 2019b; Hu et al., 2019). ES is mainly generated by wastewater treatment plant (WWTP) with large yield (Ghosh, 2009; Ali et al., 2019). It is difficult to treat ES, and its post-processing cost is relatively high (Hossain et al., 2018). Moreover, secondary pollution might easily happen if ES is not properly treated (Sun et al., 2018). Kanterli reported that sludge-based biochar (SBB) showed high sorption capacity (112.40 mg/g) for Cr(VI) (Ismail Cem and Jale, 2009). SBB prepared from municipal sludge (11.27 mg/g) and papermaking sludge (11.78 mg/g) by hydrothermal treatment had good sorption capacity for Pb(II) removal, too (Alatalo et al., 2013). In addition, SBB and Fe_3_O_4_-modified SBB also showed high uranium ion sorption efficiency (more than 90.0%) (Zeng et al., 2020; Guanhai et al., 2021). What is more, the treatment of UCW by SBB cannot only effectively solve the problem from ES, but also achieve the effect of waste treatment fee and waste resource utilization. However, to make SBB more practical, its sorption capacity for uranium or other heavy metals needs to be further improved.

So far, the most effective method for improving biochar sorption capacity or removal rate of heavy metals is to increase specific surface area or functional groups on its surface. For example, the effect of nitric acid on the surface area enlargement of biochar has been reported (Ioanna et al., 2017; Mishra et al., 2017). In addition, oxygen functional groups (Anirudhan and Deepa, 2015), humus (Zong et al., 2015), amine (Zhao et al., 2015), amino amine (Deb et al., 2012), dopaminer (Wu et al., 2017), and oximer (Xiong et al., 2017) were considered as corresponding functional groups to improve heavy metal removal rate. –COOH, as a representative of oxygen functional groups, is suitable for the complexation of uranium ions (Park et al., 2019). However, there is still a lack of research on the simultaneous expansion of pores and the addition of groups to recover more uranium ions in SBB.

In order to achieve the above requirements, the removal and recovery efficiency of uranium from UCW was comprehensively studied by involving acetic acid-modified SBB (ASBB) prepared from ES and acetic acid, including 1) differences in uranium recovery efficiency from UCW when SBB or ASBB were used, 2) impacts of variety factors (reaction time, pH, dosage, initial concentration, desorption, and interfering ions) on uranium removal by ASBB, 3) kinetic and thermodynamic analysis of sorption, 4) ASBB uranium removal mechanism based on Brunauer–Emmett–Teller (BET), scanning electron microscopy (SEM), energy-dispersive x-ray spectroscopy (EDS), Fourier-transform infrared spectroscopy (FTIR), and x-ray photoelectron spectroscopy (XPS) techniques. In this paper, acetic acid was used as a modifier to modify SBB and to treat UCW. This modification method could also be used to treat other heavy metal ions in the future.

## Materials and Methods

### Starting Materials

ES was obtained from a WWTP located in Hengyang, China. The reagents used in this study were of analytical grade. Chloroacetic acid (CH_2_ClCOOH), hydrochloric acid (HCl), ferrous sulfate heptahydrate (FeSO_4_.7H_2_O) and sodium hydroxide (NaOH), potassium hydroxide (KOH), and acetic acid (CH_3_COOH) were purchased from Sinopharm Group Pharmaceutical Co., Ltd. (Shanghai). Arsenio III [(HO)_2_C_10_H_2_(SO_3_H)_2_(N=NC_6_H_4_AsO_3_H_2_)_2_] and triuranium octoxide (U_3_O_8_) were purchased from Tianjin Kemio Chemical Reagent Co., Ltd., and China Academy of Metrology, respectively.

Uranium stock solution (1.0 g/L) was prepared by dissolving U_3_O_8_ in concentrated nitric acid. The specific preparation process was as follows: First, the dried 1.1792 g U_3_O_8_ powder was accurately weighed into a 100-ml beaker. Second, 10.0 ml of hydrochloric acid solution with a density of 1.18 g/cm³, 3.0 ml of 30 wt% hydrogen peroxide, and two drops of 1.0 mg/L of nitric acid solution were sequentially added to the beaker. Then the beaker was covered with a lid for 3 min. After time had elapsed, the solution was stirred by a glass rod for several minutes. After a violent reaction was completed, the beaker was moved a the graphite heating plate for heating and dissolution. When the dissolution was completed, the solution was cooled to room temperature. Finally, the solution was transferred to a 1,000.0-ml volumetric flask, and a nitric acid solution with pH <2 was used for constant volume. More information in detail could be found in cited literature (Lu et al., 2018a). All concentrations of UCW solutions used in the experiment were diluted by 1.0 g/L uranium stock solution.

### Sludge-Based Biochar and Acetic Acid-Modified SBB Preparation

**FIGURE 1:**
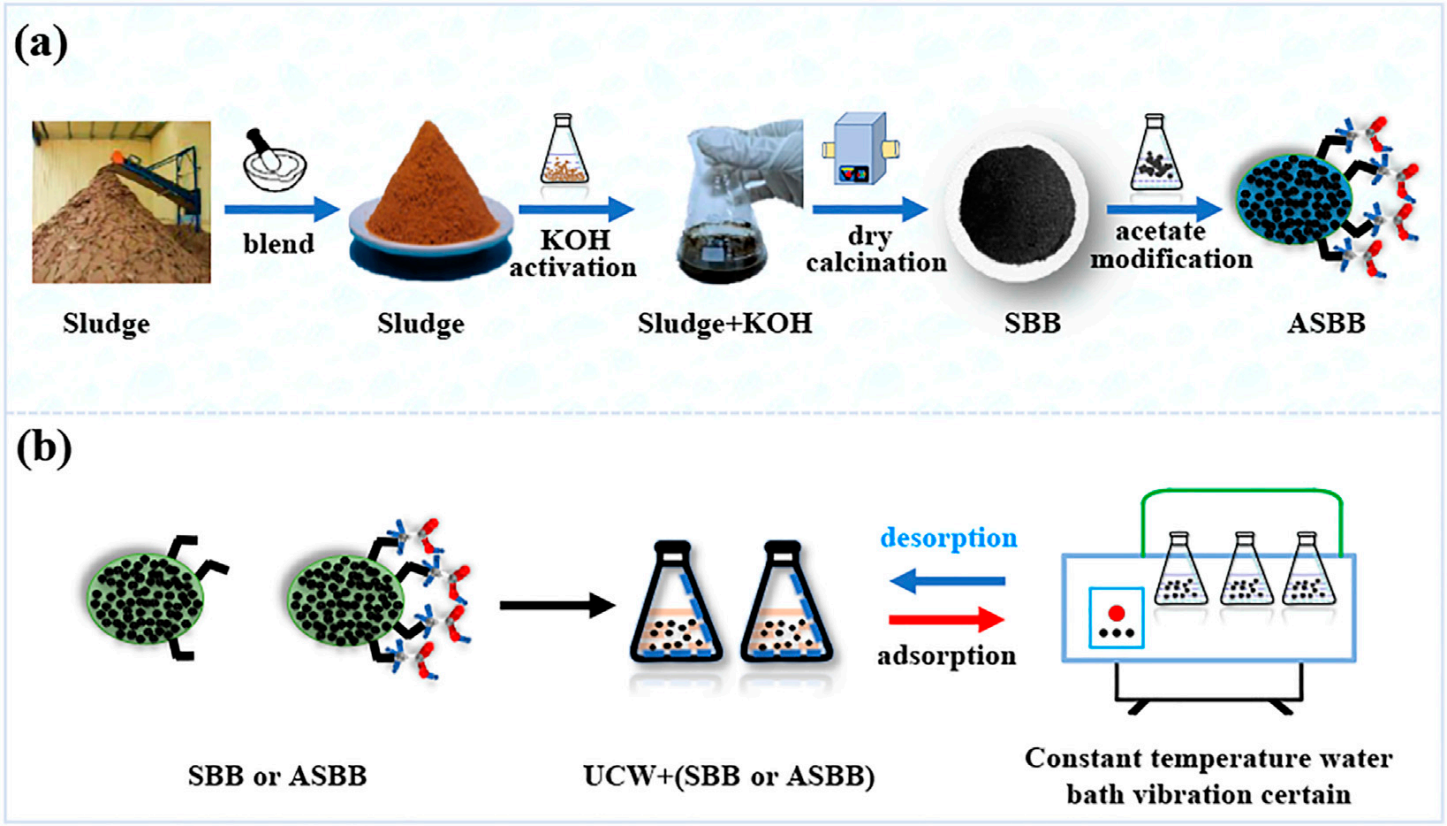
Preparation of sludge-based biochar (SBB) and acid-modified sludge-based biochar (ASBB) and uranium recovery from uranium-containing wastewater (UCW). **(A)** Preparation of SBB and ASBB. **(B)** Uranium recovery from UCW by SBB or ASBB.

The preparation process of SBB and ASBB is shown in Figure 1A. The dewatered ES was collected from WWTP and then dried at 105°C for 24 h. The dried ES was impregnated with KOH (3.0 mol/L) in proportion to mass and activated for 24 h. The impregnated ES was again dried at 80°C in a constant temperature drying oven. Thereafter, it was pyrolyzed to biochar in a muffle furnace at 350°C–700°C for 40–50 min under nitrogen atmosphere. Biochar was cooled down to room temperature under nitrogen atmosphere. Then it was washed to neutral by distilled water. The production rate of fresh SBB was 86.0 ± 10.0%. The biochar was immersed in CH_3_COOH solution (36.0%–38.0%) for 6 h and then washed with distilled water to neutrality, thus, eventually getting ASBB.

### Experimental setup

#### Orthogonal Experiments

Figure 1B displays the rationale of the experiments of uranium recovered from UCW with different SBB or ASBB dosages. A certain amount of SBB or ASBB was added to 100.0 ml of U(VI) solution. Temperature and stir speed were kept at 25°C and 120 r/min, respectively. The removal efficiencies of U(VI) by fresh SBB and ASBB were investigated according to orthogonal experiments. They were conducted under different mass ratios of sludge/KOH (MSK), calcination temperature (CTE), calcination time (CTI), and activation time (AT) (Table 1). Initial U(VI) concentration in UCW was 10.0 mg/L, pH was 3.03, and the dosage of fresh SBB or ASBB was 0.50 g/L in each investigated case.

**TABLE 1:** Effect of biochar on uranium-containing wastewater (UCW) treatment under different preparation conditions.

Influencing factors	Sludge: KOH	Calcination temperature (°C)	Calcination time (min)	Activation time (h)	Removal rate (%)		Sorption capacity (mg/g)	
	MSK	CTE	CTI	AT	SBB	ASBB	SBB	ASBB
Exp 1	3:1	400	30	3	23.6	42.8	4.72	8.56
Exp 2	2:1	400	40	6	28.1	52.2	5.62	10.44
Exp 3	1:1	400	50	12	39.3	67.3	7.86	13.46
Exp 4	1:2	400	60	24	39.9	68.9	7.98	13.78
Exp 5	1:3	400	70	48	40.8	70.8	8.16	14.16
Exp 6	3:1	450	40	12	30.6	48.6	6.12	9.72
Exp 7	2:1	450	50	24	38.7	58.3	7.74	11.66
Exp 8	1:1	450	60	48	45.2	75.1	9.04	15.02
Exp 9	1:2	450	70	3	46.3	76.2	9.26	15.24
Exp 10	1:3	450	30	6	45.1	77.5	9.02	15.5
Exp 11	3:1	500	50	48	35.1	51.2	7.02	10.24
Exp 12	2:1	500	60	3	43.1	63.1	8.62	12.62
Exp 13	1:1	500	70	6	52.2	83.1	10.44	16.62
Exp 14	1:2	500	30	12	52.5	83.6	10.5	16.72
Exp 15	1:3	500	40	24	53.2	84.1	10.64	16.82
Exp 16	3:1	550	60	6	42.8	55.8	8.56	11.16
Exp 17	2:1	550	70	12	56.2	77.2	11.24	15.44
Exp 18	1:1	550	30	24	57.8	87.1	11.56	17.42
Exp 19	1:2	550	40	48	58.6	87.2	11.72	17.44
Exp 20	1:3	550	50	3	58.9	87.4	11.78	17.48
Exp 21	3:1	600	70	24	45.1	53.1	9.02	10.62
Exp 22	2:1	600	30	48	60.7	83.9	12.14	16.78
Exp 23	1:1	600	40	3	61.2	87.9	12.24	17.58
Exp 24	1:2	600	50	6	61.6	88.1	12.32	17.62
Exp 25	1:3	600	60	12	62.3	87.9	12.46	17.58
F	87.09	9.84	4.34	1.18				
P	<0.0001	0.0106	0.0638	0.3034				

Note. The F value represents the significance of the whole fitting equation, and the larger the F implies the more significant the equation, and the better the fitting degree. *p*-Value is a parameter used to determine the hypothesis test results. The smaller the *p*-value means the more significant the result. KOH, potassium hydroxide; SBB, sludge-based biochar; ASBB, acid-modified sludge-based biochar; AT, activation time; CTI, calcination time; CTE, calcination temperature; MSK, mass ratio of sludge/KOH.

#### Batch Experiment

Several values of reaction time (1.0, 2.0, 3.0, 4.0, 5.0, 10.0, 20.0, and 30.0 min), initial pH (3.0–9.0 with a minimum interval of 1.0), adsorbent dosage (0.05, 0.1, 0.1, 0.3, 0.4, and 0.5 g/L), and initial uranium ion concentration (5.0, 10.0, 20.0, 30.0, 50.0, and 100.0 mg/L) were scrutinized. U(VI) concentration in artificial UCW was kept at 10.0 mg/L except for particular cases. Dosage of SBB or ASBB was 0.30 g/L, and pH was 6.0.

The desorption of ASBB was carried out by utilizing HCl (2.0 mol/L) as a desorption agent. The interference test of the sorption of U(VI) by coexisting ions (cation) in the solution was also carried out. Except for the solution containing 10.0 mg/L of U(VI), the concentration of coexisting ions in each solution was simulated to 10.0 mg/L. The interfering ions involved were Fe^3+^, Na^+^, Mg^2+^, Pb^2+^, and Cr^6+^. During the test, two dosages (0.3 and 0.5 g/L) of ASBB were set. HCl (0.01 mol/L) and NaOH (0.01 mol/L) were used for adjusting pH of artificial UCW.

### Analysis and Characterization

U(VI) concentration was determined by Arsenazo III spectrophotometer (Ding et al., 2018). The absorbance of UCW was measured at a wavelength of 652 nm after preheating the spectrophotometer for 30 min. Inductively coupled plasma-mass spectrometry (ICP-MS) was used to double check the values obtained from spectrophotometry. The difference in the results obtained with the two methods was 1.47%–1.53%, indicating that spectrophotometry was a reliable method under these operating conditions.

The uranium equilibrium specific sorption capacity *q*_*e*_ (mg/g) and removal rate *η* for each sorbent (SBB or ASBB) were calculated according to Eqs. 1 and 2, respectively (Liu et al., 2018):

$$q_{e}=\frac{\nu \left(c_{0}-c_{e}\right)}{m}$$ (1)

$$\eta =\frac{c_{0}-c_{e}}{c_{e}}$$ (2)

where *c*_*0*_ and *c*_*e*_ are the initial and equilibrium concentrations of uranium in the solution, *v* is the solution volume, and *m* is the mass of adsorbent. Langmuir and Freundlich sorption isotherm models were introduced to fit the U(VI) sorption data for ASBB under equilibrium conditions (Eqs 3, 4). The equations read, respectively (Christou et al., 2019):

$$q_{e}=\frac{q_{m}K_{L}Ce}{\left(1+K_{L}Ce\right)}$$ (3)

$$q_{e}=K_{F}Ce^{\frac{1}{n}}$$ (4)

where *q*_*m*_ is the maximum specific sorption capacity, *K*_*L*_ is the Langmuir equilibrium constant (L/mg), while *K*_*F*_ and *n* are the Freundlich parameters, respectively, representing the sorption capacity and the sorption intensity.

In order to investigate in detail the U(VI) sorption process by ASBB, kinetic data were fitted by pseudo-first-order (Lagergren PFO, Eq. 5) and pseudo-second-order (Ho&McKay PSO, Eq. 6) models. The equations read, respectively (Li et al., 2018):

$$q_{t}=q_{e}\left[1-\exp\left(-\kappa_{1}t\right)\right]$$ (5)

$$q_{t}=\frac{\kappa_{2}q_{e}^{2}t}{1+\kappa_{2}q_{e}t}$$ (6)

where *q*_*t*_ refers to the specific sorption capacity at *t* time, *κ*_*1*_ is the PFO sorption rate constant (min^−1^), and *κ*_*2*_ is the PSO sorption rate constant (g/mg·min^−1^).

To compare the content of acidic functional groups on the surface of SBB, ASBB, and acetic acid-modified sludge-based biochar—uranium (ASBB-U), the contents of –OH, –COO, and –COOH were determined by the Boehm method (Kalijadis et al., 2011). Three samples of 1.0 g of each material were accurately weighed, and the samples were put into 100.0-ml conical flasks. Then three samples of each material were added to 25.0 ml of 0.05 mol/L NaOH, Na_2_CO_3,_ and NaHCO_3_ standard solution, respectively. Nine samples were all stirred for a 24-h reaction and then filtered. During filtration, they were fully washed with distilled water. All the filtrates were collected independently. Methyl red was used as the end indicator of the filtrate. The unreacted alkali in the filtrate was titrated to end by a standard solution of 0.05 mol/L of HCl. The content of –OH, –COO, and –COOH was calculated by the amount of HCl.

The existing forms of uranium in UCW (10.0 mg/L) and P_CO2_ = 10^–3.5^ atm under pH from 3.0 to 9.0 were simulated by Visual MINTEQ 3.1 (Schierz and Zänker, 2009; Zong et al., 2017). The specific surface area of fresh or used SBB and ASBB was determined by BET technique (TriStar II Plus 2.02, Micromeritics, USA). The morphology of fresh or used SBB and ASBB was characterized by SEM (JSM-7500F, JEOL, JPN) coupled with EDS (INCA, Oxford, USA). Functional groups on fresh or used SBB and ASBB were analyzed through FTIR (Nicolet-iS50, Thermo Fisher Scientific, USA). The composition and chemical states of ASBB after UCW sorption were examined by XPS (Escalab 250Xi, Thermo Fisher Scientific, United States) with AlΚα radiation. The binding energies were calibrated by using containment carbon (C1s = 284.7 eV). The data analysis was carried out *via* Casa XPS software (Version 2.3.13).

## Results and Discussion

### Comparison Between Sludge-Based Biochar and Acetic Acid-Modified Sludge-Based Biochar in Uranium-Containing Wastewater Sorption

**FIGURE 2:**
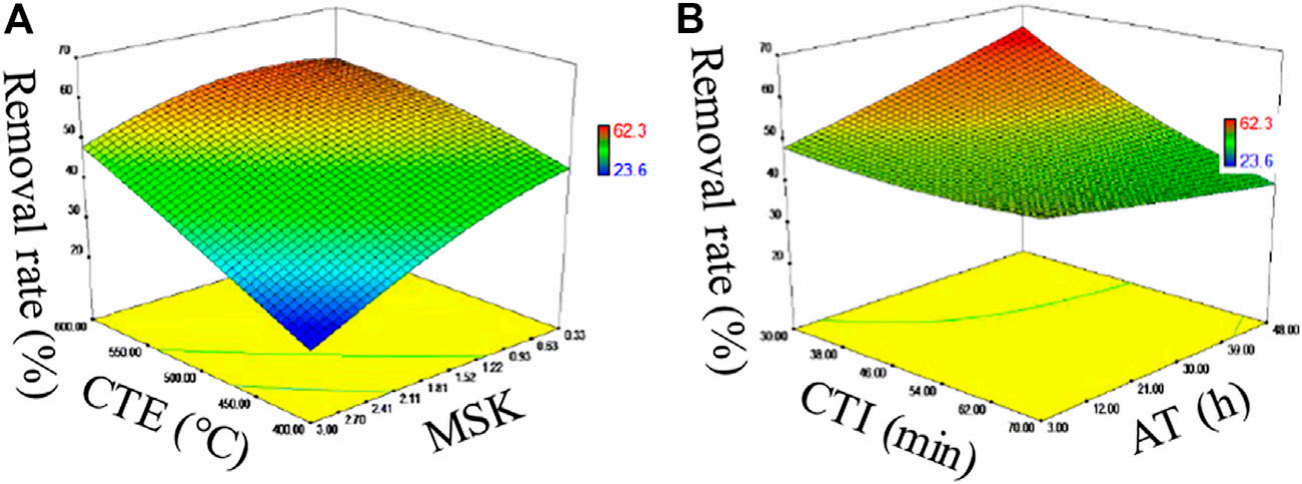
**(A)** Uranium sorption from SBB and ASBB impacted by calcination temperature (CET) and sludge: KOH (MSK), **(B)** Uranium sorption from SBB and ASBB impacted by calcination time (CTI) and activation time (AT).

The performance of U(VI) removal is presented in Figure 2. The removal rate of U(VI) by fresh SBB and ASBB gradually increased with the decrease in MSK and with the increase in CTE (Figure 2A), CTI (Figure 2B), and AT. The *F* values of MSK, CTE, CTI, and AT were 87.09, 9.84, 4.34, and 1.18, respectively, (refer to Table 1). The *p*-values were <0.0001, 0.0106, 0.0638, and 0.3034, respectively. These results indicate that the influence ranking of the explored parameters is MSK, CTE, CTI, and AT. In particular, MSK had an extremely significant effect, and CTE showed a similar tendency (Anna et al., 2018). In addition, the removal rate and sorption capacity of ASBB were higher than SBB, indicating that acetic acid modification of the biochar showed excellent effect on U(VI) removal. Altogether, the optimal preparing conditions for fresh SBB and ASBB are suggested as: MSK = 1:1, CTE = 550°C, CTI = 30 min, and AT = 24 h.

### U(VI) Removal Efficiencies by Acetic Acid-Modified Sludge-Based Biochar Under Different Conditions

#### Reaction Time

**FIGURE 3:**
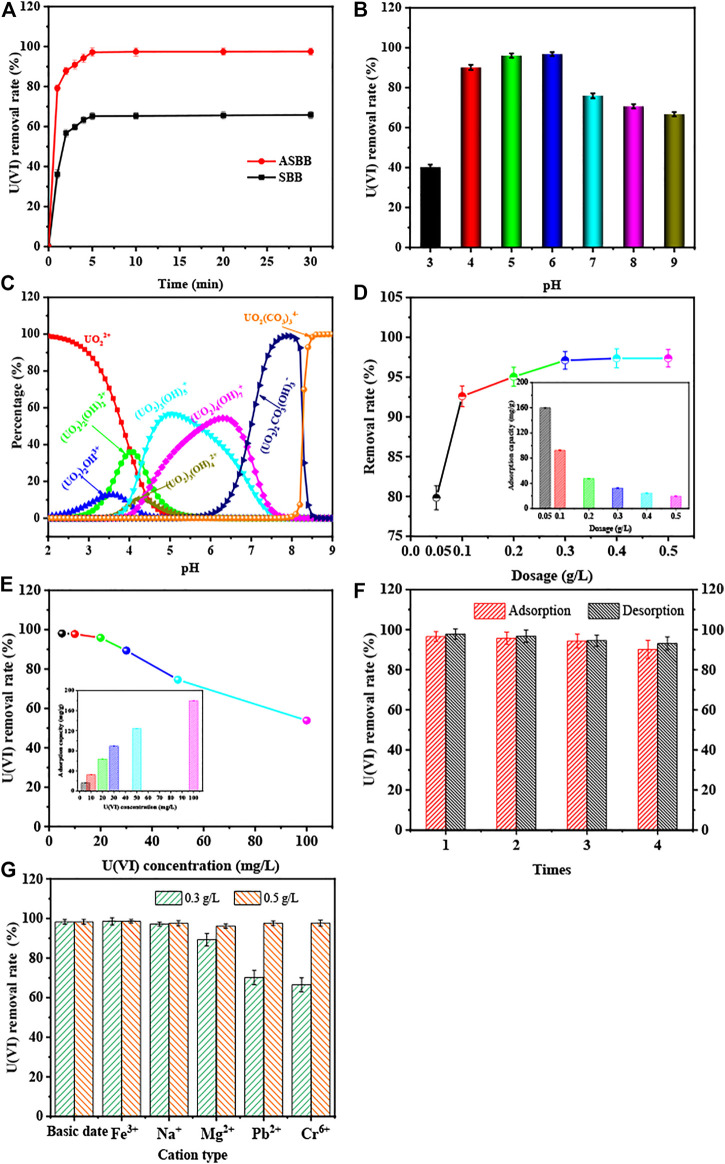
Sorption rate of U(VI) by SBB or ASBB under different conditions. **(A)** Reaction time of SBB or ASBB for U(VI) sorption, **(B)** U(VI) sorption by ASBB under difference initial pH of USW, **(C)** simulation calculation of the existing state of uranium ions under different pH conditions, **(D)** removal rate of uranium in USW by ASBB under different dosage, **(E)** the removal rate of uranium ions by ASBB at different initial concentrations of USW, **(F)** desorption efficiency of uranium ions by ASBB, **(G)** effect of interfering ions on sorption of uranium ions by ASBB in USW.

Figure 3A depicts the removal rate of U(VI) as a function of time for SBB and ASBB. The removal rate of U(VI) by SBB and ASBB increased with time quickly, and the sorption equilibrium was practically achieved within 5.0 min. This phenomenon was mainly due to the high U(VI) concentration, and to the large number of sorption sites made available by SBB and ASBB. U(VI) could rapidly diffuse to the adsorbent particle due to the high concentration gradient, to be then adsorbed on the solid surface-active sites. However, the removal rate and sorption capacity of U(VI) were close to the peak after 5.0 min. Two main reasons could explain this observation. First, the U(VI) concentration in the solution was quite low, and the U(VI) concentration was considered as one main limiting factor for the improvement of U(VI) removal rate. Second, the surface sorption sites decreased as the reaction proceeds. The probability of U(VI) binding to sorption sites was then decreased. As shown in Figure 3A, the U(VI) removal rate by SBB and ASBB was 62.8% and 97.8%, respectively. Meanwhile, the specific sorption capacity of these two adsorbents was 20.9 and 32.6 mg/g, respectively. The sorption capacity of U(VI) by ASBB was 55.8% higher than that of SBB. These results showed that ASBB could adsorb U(VI) more rapidly and efficiently, and the sorption equilibrium could be achieved within 5.0 min.

#### Initial pH of Aqueous Solution

Figures 3B, C illustrate the experimental and simulation results of the influences of the initial pH value of the aqueous solution. Figure 3B shows that sorption of U(VI) from ASBB was greatly influenced by pH. Figure 3C displays that the existing uranium morphology varies under different pH conditions. The main morphologies were UO_2_^2+^, (UO_2_)_2_(OH)_2_^2+^, (UO_2_)_2_OH^3+^, (UO_2_)_3_(OH)_5_^+^, (UO_2_)_3_(OH)_4_^2+^, (UO_2_)_4_(OH)_7_^+^, UO_2_(CO_3_)_3_^4−^, and (UO_2_)_2_CO_3_(OH)_3_^−^. The U(VI) removal rate was only 42.4% when pH = 3.0, where uranium mainly exists in the form of UO_2_^2+^ in UCW. Because the solution pH value was in this case too low, a lot of H^+^ competed with UO_2_^2+^ sorption. Meanwhile, an H^+^ proton layer on the surface of ASBB could be formed, rather than UO_2_^2+^. The electrostatic repulsion of ASBB to UO_2_^2+^ might increase; thus, the removal rate of uranium was relatively low (Wu et al., 2019). When the pH was between 4.0 and 6.0, the uranium in solution mainly existed in the form of (UO_2_)_2_(OH)_2_^2+^, (UO_2_)_3_(OH)_5_^+^, (UO_2_)_4_(OH)_7_^+^. The low protonation degree of these forms favored the sorption of uranium by ASBB (Zhu et al., 2018). With the increase in pH, many organic functional groups (such as –OH, –COOH, etc*.*) might be gradually assembled on the surface of ASBB. H^+^ on these groups then decreased, so the electronegativity of these groups increased. The binding ability and reaction probability between functional groups and uranium increased due to this phenomenon. The uranium removal rate increased under this condition. When pH was 6.0, the U(VI) removal rate peak was 97.2%. When pH was between 7.0 and 9.0, the uranium was mainly in the form of UO_2_(CO_3_)_3_^4−^ and (UO_2_)_2_CO_3_(OH)_3_^−^. These forms were difficult to be adsorbed by ASBB, and the removal rate of uranium was reduced. Therefore, pH = 6.0 was suggested as the optimal condition for U(VI) sorption from UCW by ASBB.

#### Dosage of Acetic Acid-Modified Sludge-Based Biochar

Figure 3D shows the effect of different ASBB dosages on U(VI) removal. The initial U(VI) concentration was 10.0 mg/L. The U(VI) removal rate increased from 79.8% to 97.8% when the dosage of ASBB increased from 0.05 to 0.5 g/L. With the increase in the dosing amount, the reaction sites of ASBB in UCW increased as well. The probability of U(VI) to interact with reaction sites, therefore, increased and the U(VI) removal efficiency was improved. In general, 0.30 g/L was determined as the optimal dosage used in further sections also taking into account economic reasons.

#### Initial U(VI) Concentration

Figure 3E illustrates the sorption capacity of ASBB and uranium removal rate under different initial U(VI) concentrations in the wastewater recovered by ASBB biochar. When the dosage of ASBB was 0.30 g/L, the UCW removal rate result is equal to 98.1% (initial concentration = 5.0 mg/L) and 97.8% (initial concentration = 10.0 mg/L). Namely, with the increase in U(VI) initial concentration, the removal rate of U(VI) by ASBB gradually decreased, while the specific sorption capacity was increased. The latter might be due to the excess U(VI) in the system, that drives the sorption process. Moreover, when the dosage of ASBB was 3.0 g/L (i.e., one order of magnitude higher), the removal efficiency for 100.0 mg/L of uranium concentration in UCW was 95.7%. These results demonstrated that ASBB was not only suitable for the uranium recovery from UCW with low uranium concentration but also for high concentration values. In addition, Table 2 shows the results for different adsorbents. The U(VI) sorption capacity of ASBB per unit time was about 10–1,000 times that of other materials, indicating that ASBB was a rapid and efficient U(VI) adsorbent, with interesting industrial perspectives.

**TABLE 2:** Comparison of the maximum sorption capacities of different adsorbents toward U(VI).

Adsorbent	U(VI) (mg/L)	Dosage (g/L)	Q_max_ (mg/g)	pH	Time (h)	q_t_/t[(mg/g)/h]	References
Fe_3_O_4_@C@ASA	4.76	0.6	46.20	4.00	24.00	1.91	Li et al. (2018)
HTC–COOH	140.0	0.5	163.00	4.50	24.00	6.79	Cai et al. (2017)
Activated carbon	200.0	2.5	45.24	6.00	5.00	9.05	Morsy and Hussein, (2011)
MAO-chitosan	480.0	1.0	117.65	6.00	5.00	23.53	Zhuang et al. (2018)
P(AO)-g-CTS/BT	100.0	2.0	49.90	8.00	1.00	49.90	Anirudhan et al. (2019)
SDACA	100.0	8.0	105.26	5.00	2.00	52.63	El-MagiedAbd et al. (2017)
PVP/CS	11.9	1.0	167.00	6.00	2.50	66.80	Christou et al. (2019)
AO-MWCNTs	10.0	1.0	67.90	5.00	1.00	67.90	Wu et al. (2018)
PAF	10.0	1.0	115.31	5.00	1.00	115.31	Saleh et al. (2017)
MWCNTs	25.0	0.1	83.40	6.25	0.67	124.45	Ebrahim et al. (2017)
P-Fe-CMK-3	20.0	0.2	150.00	4.00	0.50	300.00	Husnain et al. (2017)
ASBB	10.0	0.3	179.77	6.00	0.08	2,247.13	This work

#### Desorption From Acetic Acid-Modified Sludge-Based Biochar

The desorption performance of an adsorbent is an important standard to judge whether it can be practically used. Research has shown that adsorbed U(VI) could be replaced by H^+^ through ion exchange (Wen et al., 2016), and then dissolved in acidic solution (Tu et al., 2019). Figure 3F displays the results of uranium desorption from ASBB. It could be seen that after sorption and desorption for several cycles, the removal efficiency of uranium by ASBB remained at 90.2%, while the desorption efficiency from ASBB was 93.0%. These results showed that ASBB had good reusability potential, and the recovery of U(VI) could be achieved in practice.

#### Interfering Ions

Figure 3G shows the interference of coexisting ions on ASBB’s sorption of U(VI). When the dosage of ASBB was 0.30 g/L, Na^+^ had little effect on the removal of U(VI) by ASBB, while it would be inhibited by Mg^2+^, Pb^2+^, and Cr^6+^. In particular, Cr^6+^ had the greatest impact on ASBB’s sorption of U(VI). The main reason for this phenomenon might be the competitive sorption of these ions and U(VI) on the surface of ASBB. Unlike these ions, Fe^3+^ facilitated the U(VI) removal. The main reason might be that when pH = 6, Fe^3+^ could be hydrolyzed into Fe(OH)_3_ colloids (Feng et al., 2013), and U(VI) could be combined with Fe(OH)_3_ (Bruno et al., 1995). As a result, the efficiency of ASBB’s removal of U(VI) was improved. When the dosage of ASBB was increased to 0.50 g/L, U(VI) could still be efficiently adsorbed by ASBB under the interference of various ions. Therefore, when there are interfering ions in the solution, it is recommended to increase the dosage of ASBB or add a certain amount of Fe^3+^ to improve the removal rate of U(VI).

### Kinetic and Thermodynamic Analysis of Sorption

**FIGURE 4:**
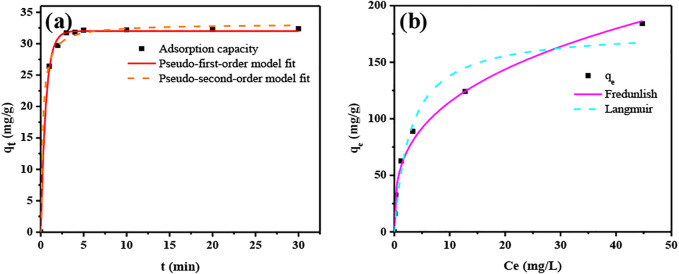
Kinetic and thermodynamic fitted curve. **(A)** Kinetic fitted curve, **(B)** thermodynamic fitted curve.

Figure 4A and Table 3 show the results of the PFO and PSO models when they were applied to experimental data. The correlation coefficient *R* ^2^ was 0.998 (PFO) and 0.997 (PSO), indicating that both physical and chemical sorption occurred during the sorption of U(VI) by ASBB.

**TABLE 3:** Kinetic parameters of U(VI) sorption on ASBB.

U(VI) concentration (mg/L)	Pseudo-first-order kinetics			Pseudo-second-order kinetics		
	*k*_*1*_/min^−1^	*q*_*e*_ */*(mg/g)	*R* ^2^	*k*_*2*_/min^−1^	*q*_*e*_ */*(mg/g)	*R* ^2^
10.0 mg/L	1.716	31.665	0.996	0.126	33.087	0.999

The results of the thermodynamic analysis are illustrated in Figure 4B and Table 4. The maximum specific sorption capacity was *q*_*m*_ = 178.194 mg/g (ASBB adsorbent), a value consistent with the laboratory result *q*_*m*_ = 179.88 mg/g. The correlation coefficient was 0.943 (Langmuir model) and 0.989 (Freundlich model), indicating that the sorption of U(VI) by ASBB was mostly dominated by multilayer sorption.

**TABLE 4:** Thermodynamic parameters of sludge-based biochar on U(VI) sorption.

Adsorbents	Langmuir			Freundlich		
	*q*_*m*_/(mg/g)	*K*_*L*_ */*(L/mg)	*R* ^2^	*K*_*F*_	*q*_*e*_ */*(mg/g)	*R* ^2^
	178.194	0.344	0.943	54.584	0.323	0.989

### Characterization and Mechanism Analysis of Uranium Recovered by Acetic Acid-Modified Sludge-Based Biochar

#### Morphological Characteristics of Sludge-Based Biochar, Acetic Acid-Modified Sudge-Based Biochar, and Acetic Acid-Modified Sludge-Based Biochar—Uranium

**FIGURE 5:**
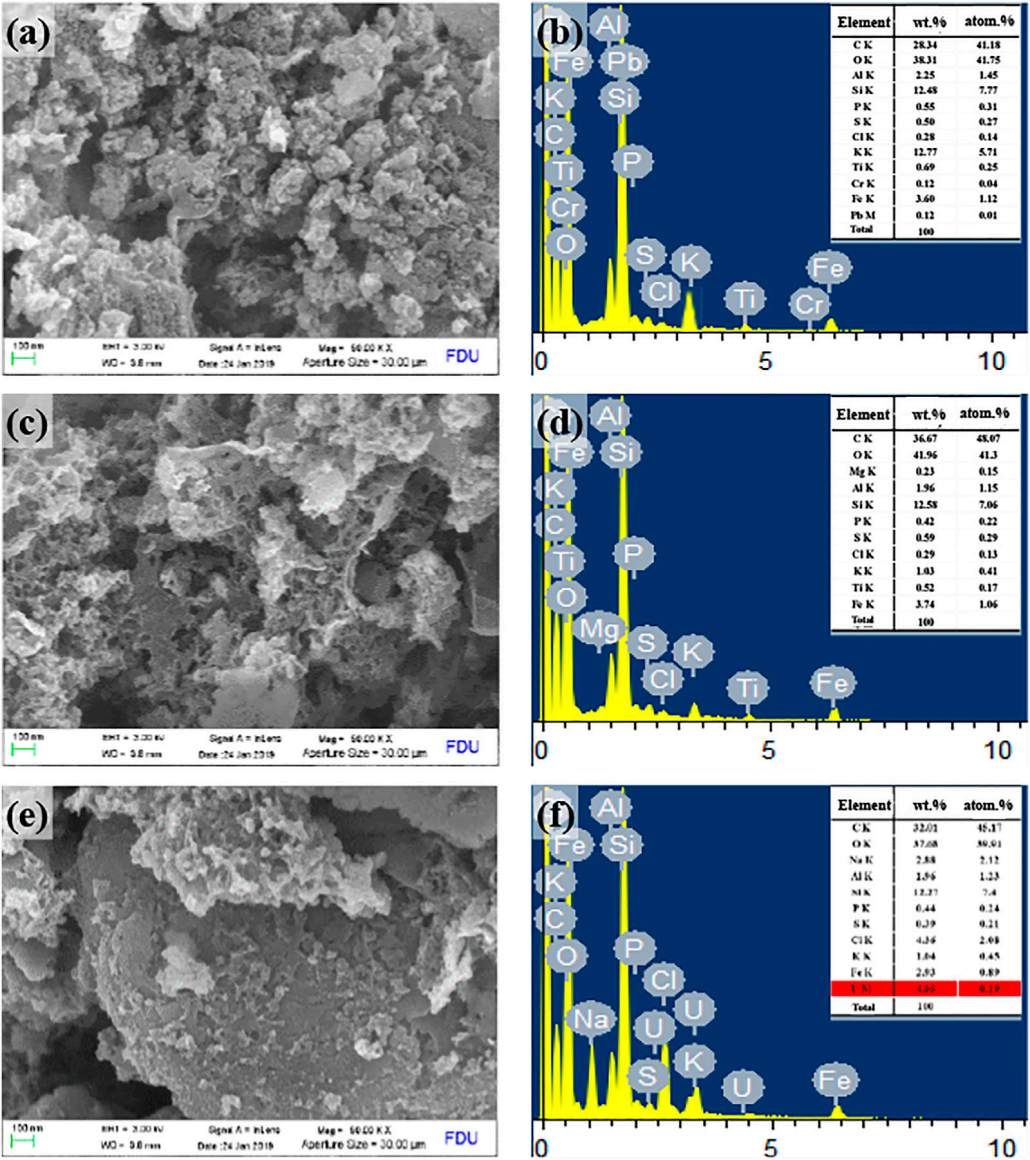
Scanning electron microscope (SEM) and energy-dispersive x-ray spectroscopy (EDS) characterization results of SBB, ASBB, and ASBB-U, **(A)** SEM of SBB’ surface, **(B)** EDS of SBB’ surface, **(C)** SEM of ASBB’ surface, **(D)** EDS of ASBB’ surface, **(E)** SEM of ASBB-U’ surface, **(F)** EDS of ASBB-U’ surface.

The microstructure and surface elements of SBB, ASBB, and ASBB-U (i.e., used ASBB adsorbent after uranium sorption) were characterized by SEM and EDS (Figure 5). As shown in Figures 5A, C, E, the pore size of the SBB surface was quite small, while a more developed pore structure was presented on the surface of ASBB. More reaction sites could be provided by ASBB to adsorb U(VI). When the sorption was completed, the ASBB microstructure changed. The pore structure of ASBB-U obviously decreased, due to the combination of U(VI) with the functional groups on the ASBB surface, or to the direct sorption of U(VI) in the pore network of ASBB. These results were consistent with the results of BET analysis (vide infra).

According to Figure 5B, the main surface elements of SBB were C, O, K, and Si in general. Figure 5D shows that the fresh ASBB surface mainly consisted of C, O, Al, Si, P, K, and Fe. An amount of U was observed on the ASBB-U surface (Figure 5F). The weight percentage was about 4.05 wt%. This indicated that uranium was successfully adsorbed by ASBB.

#### Brunauer–Emmett–Teller Comparison of Sludge-Based Biochar, Acetic Acid-Modified Sludge-Based Biochar, and Acetic Acid-Modified Sludge-Based Biochar—Uranium

**FIGURE 6:**
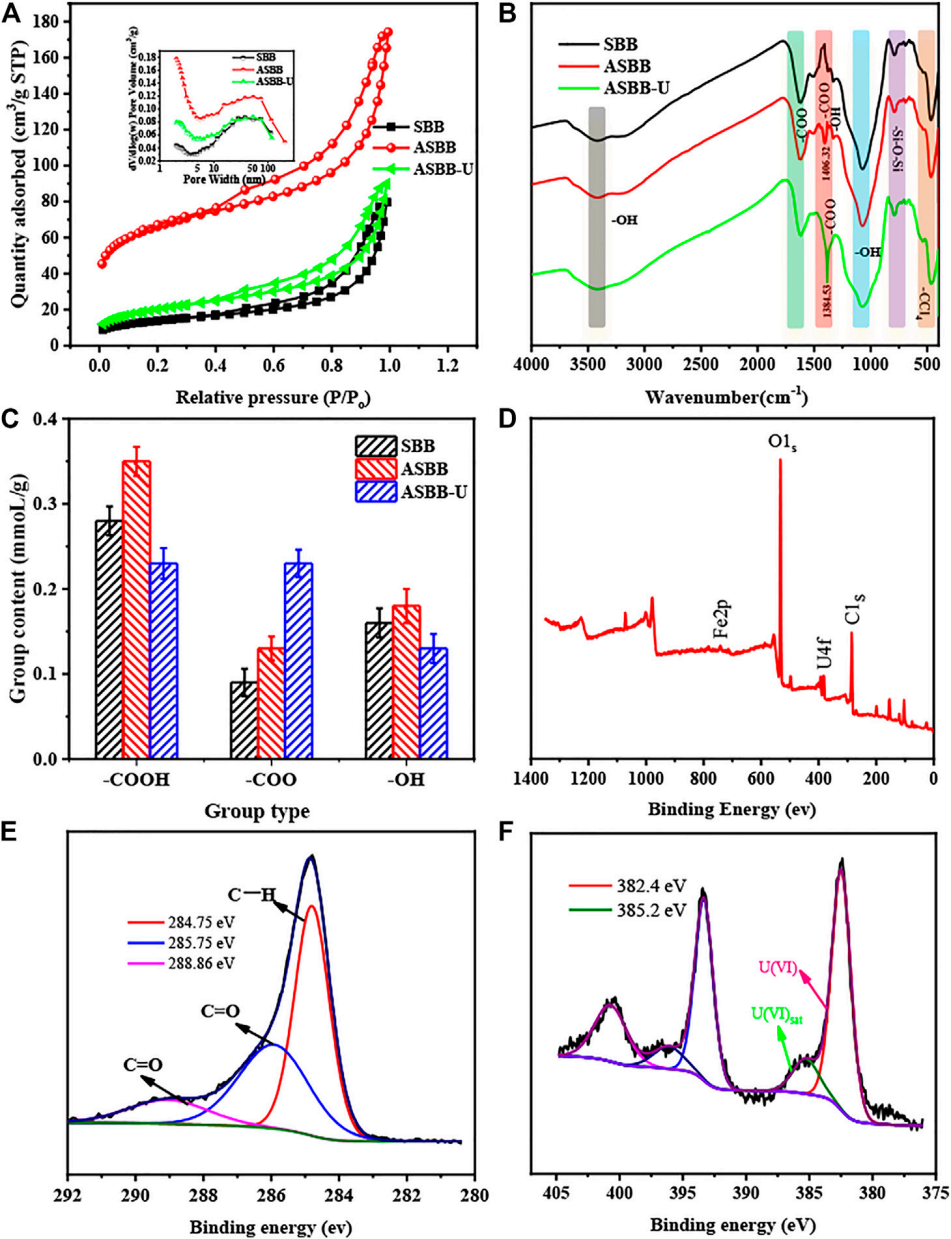
Characteristics of SBB, ASBB, and acetic acid-modified sludge-based biochar—uranium (ASBB-U). **(A)** Brunner–Emmet–Teller (BET), **(B)** Fourier transform infrared spectroscopy (FTIR), **(C)** acidic group content, **(D)** x-ray photoelectron spectroscopy (XPS) total survey scans of ASBB-U, **(E)** XPS spectra of C1s, **(F)** XPS spectra of U4f.

Figure 6A and Table 5 show the BET results for SBB, ASBB, and ASBB-U. As in Figure 6A, the isothermal sorption–desorption curves of SBB, ASBB, and ASBB-U all belonged to the unique I/IV isothermal sorption–desorption path with the H_4_ hysteresis curve (Lu et al., 2018b). It means that the porosity network of these materials was structured into micropores, mesopores, and macropores. According to the pore size distribution map (Figure 6A inside), SBB was mainly mesoporous and macroporous (mean pore size around 50 nm), ASBB was mainly mesoporous (pores of 2 and 20–50 nm), and ASBB-U was mainly mesoporous (2 nm pores) and meso/macroporous (50 nm pores). By comparing the pore size distribution of ASBB before and after uranyl ion sorption, it was found that mesopores decreased after uranium sorption, indicating that the main reaction site was within this pore range. In addition, an inflection point near the monolayer sorption was observed in the isotherm. Multilayer sorption gradually took place with the increase in relative pressure. These phenomena were consistent with the fitting results by the sorption isotherm models above.

**TABLE 5:** Surface aperture analysis.

Sample	SSA (m^2^/g)	Average pore width (nm)	Volume (cm^3^/g)
SBB	49.26	10.00	0.12
ASBB	241.42	8.35	0.21
ASBB-U	72.52	7.67	0.14

Note. SSA, specific surface area; ASBB-U, acetic acid modified sludge-based biochar—uranium.

The specific surface area (SSA) of ASBB increased with respect to the untreated biochar. Namely, a pore expansion function of acetic acid was observed. Then SSA for ASBB-U decreased. It indicates that uranyl ion was adsorbed in the pores of the ASBB surface. When the pores were blocked by the absorbed uranyl ion, the SSA of the adsorbent obviously decreased.

#### Group analysis of Sludge-Based Biochar, Acetic Acid-Modified Sludge-Based biochar, and Acetic Acid-Modified Sludge-Based Biochar-Uranium

FTIR analysis allowed to investigate the functional group modification when SBB was treated by acetic acid to give ASBB and the interaction of these groups with uranium during the sorption of U(VI) on ASBB. Results are illustrated in Figure 6B. According to literature (Gulnaz et al., 2005; Weng, 2010; Meng et al., 2019), –OH (3,427 and 1,070 cm^−1^), –COO (1,406 and 1,617 cm^−1^), –Si–O–Si (781 cm^−1^), and -CCl_4_ (476 cm^−1^) were the main groups retrieved on the surface of SBB, ASBB, and ASBB-U. When the FTIR spectra of SBB, ASBB, and ASBB-U are compared, it is seen that –COO (1,406.32 cm^−1^) was found on the ASBB surface as a new group with respect to SBB, indicating the modification of SBB by acetic acid. Moreover, when uranium was adsorbed on ASBB, some of the peak’s position and intensity changed. The peak of –OH stretching vibration at 1,331.37 cm^−1^ disappeared, indicating that –OH might react with U(VI) by deprotonation. In addition, the symmetric stretching vibration peak of –COO at 1,406.32 cm^−1^ moved to 1,384.53 cm^−1^. Although the peak shape was stable, its intensity was enhanced. The difference of the stretching vibration frequency between –COO antisymmetric stretching vibration peak (1,617.82 cm^−1^) and –COO symmetric stretching vibration peak (1,384.53 cm^−1^) was more than 200 cm^−1^ (233.29 cm^−1^). This indicates that –COO and U(VI) were combined in monodentate coordination mode (Weng, 2010).

Figure 6C shows the acid group content of SBB, ASBB, and ASBB-U. Compared with SBB, the contents of –COOH and –COO in ASBB had been increased by 0.07 and 0.04 mmol/g, respectively, indicating that SBB had been well loaded with acetic acid, and its loading was about 0.11 mmol/g. After the ASBB reaction in UCW was completed, the content of –COOH was significantly reduced, while the content of –COO was increased, indicating the sorption of –COOH on U(VI). Combined with FT-IR analysis, U(VI) could be combined with –COO to purify UCW.

#### Valence state on Acetic Acid-Modified Sludge-Based Biochar—Uranium’s Surface

Figure 6D–F present the XPS analysis results for ASBB-U’s surface. From Figure 6D, it is seen that the main peak around 532 eV belongs to O1s, the peak around 285 eV to C1s, and the peak around 382 and 375 eV to U4f. It could be concluded that the surface of ASBB-U was mainly composed of C and O elements, and a certain amount of U(VI) adsorbed on the surface. In C1s spectrum (Figure 6E), the C1s component near 284.75 eV might be associated with C–H (Ding et al., 2018). Besides, the C1s spectrum could show C = O near 285.75 and 288.86 eV (Zhao et al., 2019). Moreover, the blending energy of U4f_2/5_ (382.4 eV, 385.2 eV) corresponded to U(VI) on the surface of ASBB-U (Figure 6F) (Husnain et al., 2017; Tan et al., 2018), indicating that no redox reaction happened in uranium sorption process by ASSB.

#### Mechanism of Modification and Sorption

**FIGURE 7:**
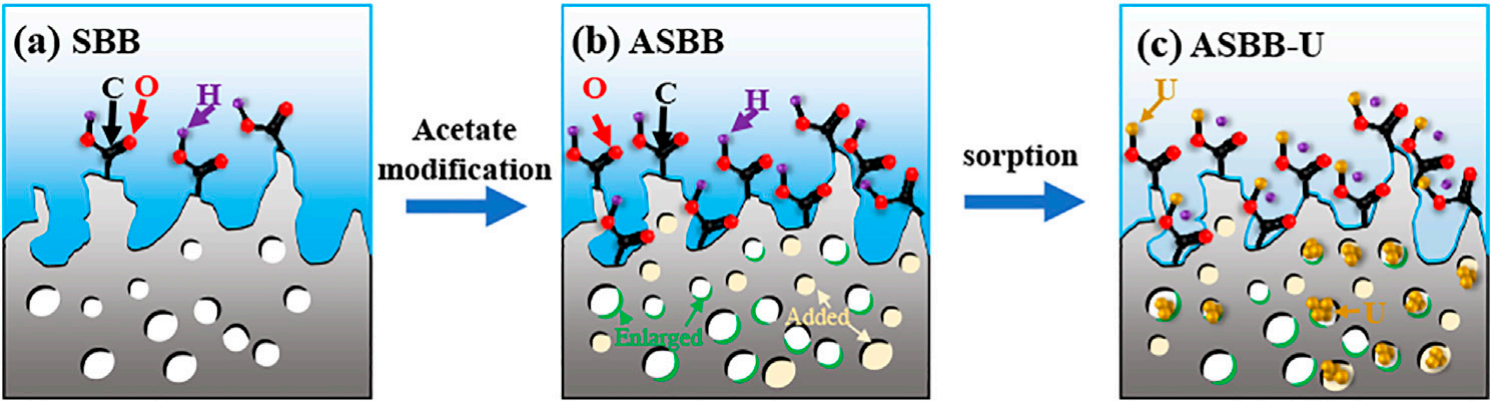
Schematic diagram of SBB modification and uranium adsorbed by ASBB. **(A)** SBB, **(B)** ASBB, **(C)** ASBB-U.

Following the experimental results, various characterization methods (BET, SEM, EDS, FTIR, XPS), and relevant references (Li et al., 2021; Liu et al., 2021), the mechanism of SBB modification and uranium sorption by ASBB was inferred. A schematic diagram is displayed in Figure 7: 1) The reaction probability of ASBB to uranium was greatly improved, due to the increased pore diameter, specific surface area, and functional group (–COOH) number by acetic acid modification of SBB. 2) The most suitable interaction between uranium ion and adsorbent under suitable reaction conditions might be of van der Waals type (Hussein et al., 2016), as witnessed by the decrease in SSA and pore size after the reaction of ASBB with USW. 3) –COOH had a good uranium sorption behavior (Park et al., 2019). At pH = 5, –H on –COOH could be easily replaced by uranium, which mainly existed in the form of (UO_2_)_3_(OH)_5_^+^ and (UO_2_)_4_(OH)_7_^+^. They were combined with –COO in monodentate coordination. The specific equation reads (Eq. 7):

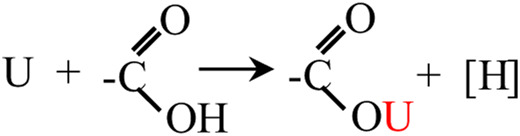

(7)



## Conclusion

Excess sludge (ES) and acetic acid were utilized to obtain a robust adsorbent starting from sludge-based biochar (SBB), for U(VI) abatement in uranium-containing wastewater (UCW). Compared with SBB, the removal efficiency and sorption capacity of the acetic acid-modified biochar (ASBB) could be effectively improved. An optimal U(VI) removal rate of 97.8% could be achieved, while initial conditions were pH = 6.0, U(VI) = 10.0 mg/L (initial concentration), adsorbent dosage = 0.30 g/L, and sorption time = 5.0 min. The beneficial effect was attributed to the double action of expanding pores and increasing –COOH functional groups following the acetic acid modification treatment. The process of U(VI) sorption by modified biochar relies on both physical and chemical sorption. The U(VI) removal mechanism by ASBB was of monodentate coordination binding between –COO– and uranium. In addition, ASBB had good reusable performance. Hence, the quick sorption and outstanding efficiency of ASBB offer a meaningful support for the use of biochar in uranium recovery from UCW and for reutilization of ES.

## Data Availability

The original contributions presented in the study are included in the article/Supplementary Material, further inquiries can be directed to the corresponding author.

## Abbreviations

ASBB, acid-modified sludge-based biochar; ASBB-U, Acetic acid-modified sludge-based biochar—uranium; BET, Brunner–Emmet–Teller; EDS, energy-dispersive x-ray spectroscopy; ES, excess sludge; FTIR, Fourier transform infrared spectroscopy; SBB, sludge-based biochar; SEM, scanning electron microscope; SSA, specific surface area; UCW, uranium-containing wastewater; WWTP, wastewater treatment plant; XPS, x-ray photoelectron spectroscopy.

## References

[B1] Abadi S. R. H. Sebzari M. R. Hemati M. Rekabdar F. Mohammadi T. (2011). Ceramic Membrane Performance in Microfiltration of Oily Wastewater[J]. Desalination 265 (1-3), 222–228. 10.1016/j.desal.2010.07.055

[B2] Abdi S. Nasiri M. Mesbahi A. Khani M. H. (2017). Investigation of Uranium (VI) Adsorption by Polypyrrole. J. Hazard. Mater. 332, 132–139. 10.1016/j.jhazmat.2017.01.013 28285106

[B3] Alatalo S. M. Repo E. Mäkilä E. Salonen J. Vakkilainen E. Sillanpää M. (2013). Adsorption Behavior of Hydrothermally Treated Municipal Sludge & Pulp and Paper Industry Sludge. Bioresour. Technol. 147, 71–76. 10.1016/j.biortech.2013.08.034 23994693

[B4] Ali Z. Chen Z. Wang X. Zhang Q. (2019). Microwave-assisted Pyrolysis of Sewage Sludge: A Review[J]. Fuel Process. Technol. 187, 84–104.

[B5] Anirudhan T. S. Deepa J. R. Binusreejayan (2015). Synthesis and Characterization of Multi-Carboxyl-Functionalized Nanocellulose/nanobentonite Composite for the Adsorption of Uranium(VI) from Aqueous Solutions: Kinetic and Equilibrium Profiles. Chem. Eng. J. 273, 390–400. 10.1016/j.cej.2015.03.007

[B6] Anirudhan T. S. Lekshmi G. S. Shainy F. (2019). Synthesis and Characterization of Amidoxime Modified Chitosan/bentonite Composite for the Adsorptive Removal and Recovery of Uranium from Seawater. J. Colloid Interf. Sci. 534, 248–261. 10.1016/j.jcis.2018.09.009 30227381

[B7] Anna C.-P. Justyna S.-B. Wojciech L. (2018). Optimization of Copper, lead and Cadmium Biosorption onto Newly Isolated Bacterium Using a Box-Behnken Design[J]. Ecotoxicology Environ. Saf. 149, 275–283. 10.1016/j.ecoenv.2017.12.008 29253787

[B8] Baur X. Rihs H.-P. Altmeyer P. Degens P. Conrad K. Mehlhorn J. (1996). Systemic Sclerosis in German Uranium Miners under Special Consideration of Autoantibody Subsets and Hla Class Ii Alleles. Respiration 63 (6), 368–375. 10.1159/000196579 8933656

[B9] Bruno J. De Pablo J. Duro L. Figuerola E. (1995). Experimental Study and Modeling of the U(VI)-Fe(OH)3 Surface Precipitation/coprecipitation Equilibria. Geochimica et Cosmochimica Acta 59 (20), 4113–4123. 10.1016/0016-7037(95)00243-s

[B10] Cai H. Lin X. Qin Y. Luo X. (2017). Hydrothermal Synthesis of Carbon Microsphere from Glucose at Low Temperature and its Adsorption Property of Uranium(VI). J. Radioanal. Nucl. Chem. 311 (1), 695–706. 10.1007/s10967-016-5106-9

[B11] Chen T. Zhang J. Ge H. Li M. Li Y. liu B. (2020). Efficient Extraction of Uranium in Organics-Containing Wastewater over G-C3n4/GO Hybrid Nanosheets with Type-II Band Structure. J. Hazard. Mater. 384, 121383. 10.1016/j.jhazmat.2019.121383 31607580

[B12] Christou C. Philippou K. Krasia-Christoforou T. Pashalidis I. (2019). Uranium Adsorption by Polyvinylpyrrolidone/chitosan Blended Nanofibers. Carbohydr. Polym. 219, 298–305. 10.1016/j.carbpol.2019.05.041 31151529

[B13] Deb A. K. S. Ilaiyaraja P. Ponraju D. Venkatraman B. (2012). Diglycolamide Functionalized Multi-Walled Carbon Nanotubes for Removal of Uranium from Aqueous Solution by Adsorption[J]. J. Radioanal. Nucl. Chem. 291 (3), 877–883.

[B14] Ding L. Tan W.-F. Xie S.-B. Mumford K. Lv J.-W. Wang H.-Q. (2018). Uranium Adsorption and Subsequent Re-oxidation under Aerobic Conditions by Leifsonia Sp. - Coated Biochar as green Trapping Agent. Environ. Pollut. 242, 778–787. 10.1016/j.envpol.2018.07.050 30031311

[B15] Ebrahim Y. S. M. Salem Nafisa A. Abdeltawab Ahmed A. (2017). Equilibrium and Thermodynamics for Adsorption of Uranium onto Potassium Hydroxide Oxidized Carbon[J]. Desalination Water Treat. 72, 335–342.

[B16] El-MagiedAbd M. O. A. Mohammaden T. F. El-Aassy I. K. Gad H. M. H. Hassan A. M. Mahmoud M. A. (2017). Decontamination of Uranium-Polluted Groundwater by Chemically-Enhanced, Sawdust-Activated Carbon. Colloids Inter. 1 (1), 2. 10.3390/colloids1010002

[B17] Fang-Zhu X. Cheng W. Li-Mei Y. Yi-Qiu P. Yu-Li X. Kang Z. (2019). Fabrication of Magnetic Functionalised Calix 4 Arene Composite for Highly Efficient and Selective Adsorption towards Uranium(VI)[J]. Environ. Chem. 16 (8), 577–586.

[B18] Feng Q. Zhang Z. Chen Y. Liu L. Zhang Z. Chen C. (2013). Adsorption and Desorption Characteristics of Arsenic on Soils: Kinetics, Equilibrium, and Effect of Fe(OH)3 Colloid, H2SiO3 Colloid and Phosphate. Proced. Environ. Sci. 18, 26–36. 10.1016/j.proenv.2013.04.005

[B19] Gerber U. Zirnstein I. Krawczyk-Bärsch E. Lünsdorf H. Arnold T. Merroun M. L. (2016). Combined Use of Flow Cytometry and Microscopy to Study the Interactions between the Gram-Negative Betaproteobacterium Acidovorax Facilis and Uranium(VI). J. Hazard. Mater. 317, 127–134. 10.1016/j.jhazmat.2016.05.062 27262280

[B20] Ghosh P. (2009). Biological Treatment Processes[J]. Handbook Environ. Eng. 80 (2), 69–72.

[B21] Guanhai M. Qing H. Guohua W. Shuibo X. Haidu N. Xiaoling Z. (2021). Fe_3_O_4_-modified Sewage Sludge Biochar for U(VI) Removal from Aqueous Solution: Performance and Mechanism[J]. J. Radioanal. Nucl. Chem. 329 (1), 225–237.

[B22] Gulnaz O. Saygideger S. Kusvuran E. (2005). Study of Cu(II) Biosorption by Dried Activated Sludge: Effect of Physico-Chemical Environment and Kinetics Study. J. Hazard. Mater. 120 (1-3), 193–200. 10.1016/j.jhazmat.2005.01.003 15811681

[B23] Hossain M. I. Paparini A. Cord-Ruwisch R. (2018). Direct Oxygen Uptake from Air by Novel Glycogen Accumulating Organism Dominated Biofilm Minimizes Excess Sludge Production. Sci. Total Environ. 640-641, 80–88. 10.1016/j.scitotenv.2018.05.292 29857323

[B24] Hu W. Xie Y. Lu S. Li P. Xie T. Zhang Y. (2019). One-step Synthesis of Nitrogen-Doped Sludge Carbon as a Bifunctional Material for the Adsorption and Catalytic Oxidation of Organic Pollutants. Sci. Total Environ. 680, 51–60. 10.1016/j.scitotenv.2019.05.098 31100668

[B25] Husnain S. M. KimJu H. J. Um W. Chang Y.-Y. Chang Y.-S. (2017). Superparamagnetic Adsorbent Based on Phosphonate Grafted Mesoporous Carbon for Uranium Removal. Ind. Eng. Chem. Res. 56 (35), 9821–9830. 10.1021/acs.iecr.7b01737

[B26] Hussein A. Fares Mohammad M. Abu Al-Rub Fahmi A. (2016). Removal of Uranium and Associated Contaminants from Aqueous Solutions Using Functional Carbon Nanotubes-Sodium Alginate Conjugates[J]. Minerals 6 (1), 9.

[B27] Ioanna L. Georgia M. Marilena D. Ioannis P. (2017). Uranium Binding by Biochar Fibres Derived from Luffa Cylindrica after Controlled Surface Oxidation[J]. J. Radioanal. Nucl. Chem. 311 (1), 871–875.

[B28] Ismail Cem K. Jale Y. (2009). Use of Waste Sludge from the Tannery Industry[J]. Energy & Fuels 23 (6), 3126–3133.

[B29] Jiang X. Wang H. Wang Q. Hu E. Duan Y. (2020). Immobilizing Amino-Functionalized Mesoporous Silica into Sodium Alginate for Efficiently Removing Low Concentrations of Uranium. J. Clean. Prod. 247, 119162. 10.1016/j.jclepro.2019.119162

[B30] Jin J. Li S. Peng X. Liu W. Zhang C. Yang Y. (2018). HNO3 Modified Biochars for Uranium (VI) Removal from Aqueous Solution. Bioresour. Technol. 256, 247–253. 10.1016/j.biortech.2018.02.022 29453051

[B31] Kalijadis A. Vukcevic M. Jovanovic Z. Lausevic Z. Lausevic M. (2011). Characterization of Surface Oxygen Groups on Different Carbon Materials by the Boehm Method and Temperature Programmed Desorption. J. Serbian Chem. Soc. 76 (5), 757–768. 10.2298/jsc091224056k

[B32] Kataria N. Garg V. K. (2018). Green Synthesis of Fe3O4 Nanoparticles Loaded Sawdust Carbon for Cadmium (II) Removal from Water: Regeneration and Mechanism. Chemosphere 208, 818–828. 10.1016/j.chemosphere.2018.06.022 29906756

[B33] Kathren R. L. Burklin R. K. (2008). Acute Chemical Toxicity of Uranium. Health Phys. 94 (2), 170–179. 10.1097/01.hp.0000288043.94908.1f 18188051

[B34] Khani M. H. Keshtkar A. R. Ghannadi M. Pahlavanzadeh H. (2008). Equilibrium, Kinetic and Thermodynamic Study of the Biosorption of Uranium onto Cystoseria Indica Algae. J. Hazard. Mater. 150 (3), 612–618. 10.1016/j.jhazmat.2007.05.010 17582680

[B35] Khawassek Y. M. Masoud A. M. Taha M. H. Hussein A. E. M. (2018). Kinetics and Thermodynamics of Uranium Ion Adsorption from Waste Solution Using Amberjet 1200 H as Cation Exchanger. J. Radioanal. Nucl. Chem. 315 (3), 493–502. 10.1007/s10967-017-5692-1

[B36] Kong L. Ruan Y. Zheng Q. Su M. Diao Z. Chen D. (2020). Uranium Extraction Using Hydroxyapatite Recovered from Phosphorus Containing Wastewater. J. Hazard. Mater. 382, 120784. 10.1016/j.jhazmat.2019.120784 31446349

[B37] Li F. Meng F. Wang H. Ge B. Zhang Y. Yu C. (2019). Urea-modified Grass Ash Activated Sludge Carbon: Structure and Adsorption Properties towards H2S and CH3SH. New J. Chem. 43 (44), 17494–17501. 10.1039/c9nj03836a

[B38] Li H. Li Y. Li B. Dai Y. Chen X. (2020). Melamine-induced Novel MSONs Heterostructured Framework: Controlled-Switching between MOF and SOF via a Self-Assembling Approach for Rapid Uranium Sequestration. Chem. Eng. J. 379, 122279. 10.1016/j.cej.2019.122279

[B39] Li M. Liu H. Chen T. Dong C. Sun Y. (2019). Synthesis of Magnetic Biochar Composites for Enhanced Uranium(VI) Adsorption. Sci. Total Environ. 651, 1020–1028. 10.1016/j.scitotenv.2018.09.259 30266047

[B40] Li M. Liu Y. Li F. Shen C. Kaneti Y. C. Yamauchi Y. (2021). Defect-Rich Hierarchical Porous UiO-66(Zr) for Tunable Phosphate Removal[J]. Environ. Sci. Technol. 55 (19), 13209–13218. 34553909 10.1021/acs.est.1c01723

[B41] Li P. Wang J. Wang X. He B. Pan D. Liang J. (2018). Arsenazo-functionalized Magnetic Carbon Composite for Uranium(VI) Removal from Aqueous Solution. J. Mol. Liquids 269, 441–449. 10.1016/j.molliq.2018.08.073

[B42] Liu F. You S. Wang Z. Liu Y. (2021). Redox-Active Nanohybrid Filter for Selective Recovery of Gold from Water. ACS EST Eng. 1 (9), 1342–1350. 10.1021/acsestengg.1c00158

[B43] Liu H. Xie S. Liao J. Yan T. Liu Y. Tang X. (2018). Novel Graphene Oxide/bentonite Composite for Uranium(VI) Adsorption from Aqueous Solution. J. Radioanal. Nucl. Chem. 317 (3), 1349–1360. 10.1007/s10967-018-5992-0

[B44] Lu B.-q. Li M. Zhang X.-w. Huang C.-m. Wu X.-y. Fang Q. (2018). Immobilization of Uranium into Magnetite from Aqueous Solution by Electrodepositing Approach. J. Hazard. Mater. 343, 255–265. 10.1016/j.jhazmat.2017.09.037 28965015

[B45] Lu S. Liu Y. Feng L. Sun Z. Zhang L. (2018). Characterization of Ferromagnetic Sludge-Based Activated Carbon and its Application in Catalytic Ozonation of P-Chlorobenzoic Acid. Environ. Sci. Pollut. Res. 25 (6), 5086–5094. 10.1007/s11356-017-8680-7 28281060

[B46] Malenchenko A. F. Barkun N. A. Guseva G. F. (1978). Effect of Uranium on the Induction and Course of Experimental Autoimmune Orchitis and Thyroiditis. J. Hyg. Epidemiol. Microbiol. Immunol. 22 (3), 268–277. 570986

[B47] Meng F. Gong Z. Wang Z. Fang P. Li X. (2019). Study on a Nitrogen-Doped Porous Carbon from Oil Sludge for CO2 Adsorption. Fuel 251, 562–571. 10.1016/j.fuel.2019.04.046

[B48] Mishra V. Sureshkumar M. K. Gupta N. Kaushik C. P. (2017). Study on Sorption Characteristics of Uranium onto Biochar Derived from Eucalyptus Wood. Water Air Soil Pollut. 228 (8), 309. 10.1007/s11270-017-3480-8

[B49] Morsy A. M. A. Hussein A. E. M. (2011). Adsorption of Uranium from Crude Phosphoric Acid Using Activated Carbon. J. Radioanal. Nucl. Chem. 288 (2), 341–346. 10.1007/s10967-011-0980-7

[B50] NaserHumood H. A. (2013). Assessment and Management of Heavy Metal Pollution in the marine Environment of the Arabian Gulf: A Review. Mar. Pollut. Bull. 72 (1), 6–13. 10.1016/j.marpolbul.2013.04.030 23711845

[B51] Park J. Bae J. Jin K. Park J. (2019). Carboxylate-functionalized Organic Nanocrystals for High-Capacity Uranium Sorbents. J. Hazard. Mater. 371, 243–252. 10.1016/j.jhazmat.2019.03.007 30852276

[B52] Pu D. Kou Y. Zhang L. Liu B. Zhu W. Zhu L. (2019). Waste Cigarette Filters: Activated Carbon as a Novel Sorbent for Uranium Removal. J. Radioanal. Nucl. Chem. 320 (3), 725–731. 10.1007/s10967-019-06502-z

[B53] Saleh T. A. Naeemullah T. M. Tuzen M. Sarı A. (2017). Polyethylenimine Modified Activated Carbon as Novel Magnetic Adsorbent for the Removal of Uranium from Aqueous Solution. Chem. Eng. Res. Des. 117, 218–227. 10.1016/j.cherd.2016.10.030

[B54] Schierz A. Zänker H. (2009). Aqueous Suspensions of Carbon Nanotubes: Surface Oxidation, Colloidal Stability and Uranium Sorption. Environ. Pollut. 157 (4), 1088–1094. 10.1016/j.envpol.2008.09.045 19010575

[B55] Sun S. Zhang S. Zhang W. Meng J. Wang L. (2018). Reduction and Heavy Metals Removal of Excess Sludge by Radio Frequency Discharge Plasma. IOP Conf. Ser. Earth Environ. Sci. 146, 012069. 10.1088/1755-1315/146/1/012069

[B56] Sun Y. Shao D. Chen C. Yang S. Wang X. (2013). Highly Efficient Enrichment of Radionuclides on Graphene Oxide-Supported Polyaniline. Environ. Sci. Technol. 47 (17), 9904–9910. 10.1021/es401174n 23902375

[B57] Sun Y. Wang X. Ai Y. Yu Z. Huang W. Chen C. (2017). Interaction of Sulfonated Graphene Oxide with U(VI) Studied by Spectroscopic Analysis and Theoretical Calculations. Chem. Eng. J. 310, 292–299. 10.1016/j.cej.2016.10.122

[B58] Tan Y. Li L. Zhang H. Ding D. Dai Z. Xue J. (2018). Adsorption and Recovery of U(VI) from Actual Acid Radioactive Wastewater with Low Uranium Concentration Using Thioacetamide Modified Activated Carbon from Liquorice Residue. J. Radioanal. Nucl. Chem. 317 (2), 811–824. 10.1007/s10967-018-5952-8

[B59] Tu H. Lan T. Yuan G. Zhao C. Liu J. Li F. (2019). The Influence of Humic Substances on Uranium Biomineralization Induced by Bacillus Sp. Dwc-2. J. Environ. Radioactivity 197, 23–29. 10.1016/j.jenvrad.2018.11.010 30502659

[B60] Wang G. Liu J. Wang X. Xie Z. Deng N. (2009). Adsorption of Uranium (VI) from Aqueous Solution onto Cross-Linked Chitosan. J. Hazard. Mater. 168 (2-3), 1053–1058. 10.1016/j.jhazmat.2009.02.157 19342166

[B61] Wen T. Wang X. Wang J. Chen Z. Li J. Hu J. (2016). A Strategically Designed Porous Magnetic N-Doped Fe/Fe3C@C Matrix and its Highly Efficient Uranium(vi) Remediation. Inorg. Chem. Front. 3 (10), 1227–1235. 10.1039/c6qi00091f

[B62] Weng Pushi. (2010). Fourier Transform Infrared Spectrum analysis[M]. Beijing, China: Chemical Industrial Press.

[B63] Wu F. Pu N. Ye G. Sun T. Wang Z. Song Y. (2017). Performance and Mechanism of Uranium Adsorption from Seawater to Poly(dopamine)-Inspired Sorbents. Environ. Sci. Technol. 51 (8), 4606–4614. 10.1021/acs.est.7b00470 28332830

[B64] Wu J. Tian K. Wang J. (2018). Adsorption of Uranium (VI) by Amidoxime Modified Multiwalled Carbon Nanotubes. Prog. Nucl. Energ. 106, 79–86. 10.1016/j.pnucene.2018.02.020

[B65] Wu Y. Chen D. Kong L. Tsang D. C. W. Su M. (2019). Rapid and Effective Removal of Uranium (VI) from Aqueous Solution by Facile Synthesized Hierarchical Hollow Hydroxyapatite Microspheres. J. Hazard. Mater. 371, 397–405. 10.1016/j.jhazmat.2019.02.110 30870644

[B66] Xiong J. Hu S. Liu Y. Yu J. Yu H. Xie L. (2017). Polypropylene Modified with Amidoxime/Carboxyl Groups in Separating Uranium(VI) from Thorium(IV) in Aqueous Solutions. ACS Sustain. Chem. Eng. 5 (2), 1924–1930. 10.1021/acssuschemeng.6b02663

[B67] Zeng T. Mo G. Zhang X. Liu J. Liu H. Xie S. (2020). U(VI) Removal Efficiency and Mechanism of Biochars Derived from Sewage Sludge at Two Pyrolysis Temperatures. J. Radioanal. Nucl. Chem. 326 (2), 1413–1425. 10.1007/s10967-020-07423-y

[B68] Zhao C. Liu J. Deng Y. Tian Y. Zhang G. Liao J. (2019). Uranium(Ⅵ) Adsorption from Aqueous Solutions by Microorganism-Graphene Oxide Composites via an Immobilization Approach. J. Clean. Prod. 236, 117624. 10.1016/j.jclepro.2019.117624

[B69] Zhao W. Lin X. Qin Y. Cai H. Chen Y. Luo X. (2017). Preparation of Chemically Oxidized Porous Carbon and its Adsorption of Uranium(VI) from Aqueous Solution. J. Radioanal. Nucl. Chem. 314 (3), 1853–1864. 10.1007/s10967-017-5559-5

[B70] Zhao Z. Li J. Wen T. Shen C. Wang X. Xu A. (2015). Surface Functionalization Graphene Oxide by Polydopamine for High Affinity of Radionuclides. Colloids Surf. A: Physicochemical Eng. Aspects 482, 258–266. 10.1016/j.colsurfa.2015.05.020

[B71] Zhu J. Liu Q. Li Z. Liu J. Zhang H. Li R. (2018). Efficient Extraction of Uranium from Aqueous Solution Using an Amino-Functionalized Magnetic Titanate Nanotubes. J. Hazard. Mater. 353, 9–17. 10.1016/j.jhazmat.2018.03.042 29627673

[B72] Zhuang S. Cheng R. Kang M. Wang J. (2018). Kinetic and Equilibrium of U(Ⅵ) Adsorption onto Magnetic Amidoxime-Functionalized Chitosan Beads. J. Clean. Prod. 188, 655–661. 10.1016/j.jclepro.2018.04.047

[B73] Zong P. Cao D. Cheng Y. Zhang H. Shao D. Wang S. (2017). Functionally Reduced Graphene Oxide Supported Iron Oxides Composites as an Adsorbent for the Immobilization of Uranium Ions from Aqueous Solutions. J. Mol. Liquids 240, 578–588. 10.1016/j.molliq.2017.05.101

[B74] Zong P. Wu X. Gou J. Lei X. Liu D. Deng H. (2015). Immobilization and Recovery of Uranium(VI) Using Na-Bentonite from Aqueous Medium: Equilibrium, Kinetics and Thermodynamics Studies. J. Mol. Liquids 209, 358–366. 10.1016/j.molliq.2015.05.052

